# Ring-whizzing in polyene-PtL_2_ complexes revisited

**DOI:** 10.3762/bjoc.12.135

**Published:** 2016-07-07

**Authors:** Oluwakemi A Oloba-Whenu, Thomas A Albright, Chirine Soubra-Ghaoui

**Affiliations:** 1 Department of Chemistry, University of Lagos, Akoka, Yaba, Lagos, Nigeria; 2 Department of Chemistry, University of Houston, Houston, Texas 77204-5003, USA; 3 Department of Chemistry and Physics, University of St. Thomas, Houston, Texas 77006, USA

**Keywords:** d^10^ metal complexes, density functional theory (DFT), hapototropic rearrangements, HOMO–LUMO interactions, polyene-ML_2_ complexes, ring-whizzing

## Abstract

Ring-whizzing was investigated by hybrid DFT methods in a number of polyene–Pt(diphosphinylethane) complexes. The polyenes included cyclopropenium^+^, cyclobutadiene, cyclopentadienyl^+^, hexafluorobenzene, cycloheptatrienyl^+^, cyclooctatetraene, octafluorooctatetraene, 6-radialene, pentalene, phenalenium^+^, naphthalene and octafluoronaphthalene. The HOMO of a d^10^ ML_2_ group (with b_2_ symmetry) interacting with the LUMO of the polyene was used as a model to explain the occurrence of minima and maxima on the potential energy surface.

## Introduction

**Figure 1:**
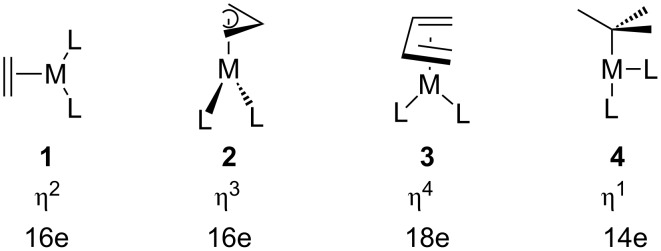
The four coordination geometries for d^10^ polyene-ML_2_ complexes along with their hapto numbers and electron count.

Polyene–transition metal complexes were found to undergo fluxional rearrangements as early as 1956 with the preparation of Cp_2_Fe(CO)_2_ [1]. The migration of an ML*_n_* unit around the periphery of a cyclic polyene is commonly called ring-whizzing, purportedly ascribed to Rowland Pettit [2]. A more inclusive term is haptotropic rearrangement [3] wherein a metal atom changes its hapticity along the reaction path. Haptotropic rearrangements in ML_3_ and MCp complexes are numerous [4–9] and have found use in synthetic strategies [10], switching devices [11–13] and energy storage [14–15]. Much less is known about the polyene–ML_2_ analogs. There are two classes of compounds; one set consists of d^8^ ML_2_ compounds [16–19] and the other, which we will be concerned with, are the d^10^ ML_2_ class. There is ample precedent for four basic coordination geometries exhibited by these compounds. These are shown in Figure 1. Notice that in each case the orientation of the ML_2_ unit is tied to the coordination number of the polyene and total electron count. One of us undertook a theoretical survey of these compounds at the extended Hückel level a number of years ago [20–21]. In the present contribution we shall revisit some of these rearrangements using DFT theory, as well as, investigate some new compounds.

**Figure 2:**
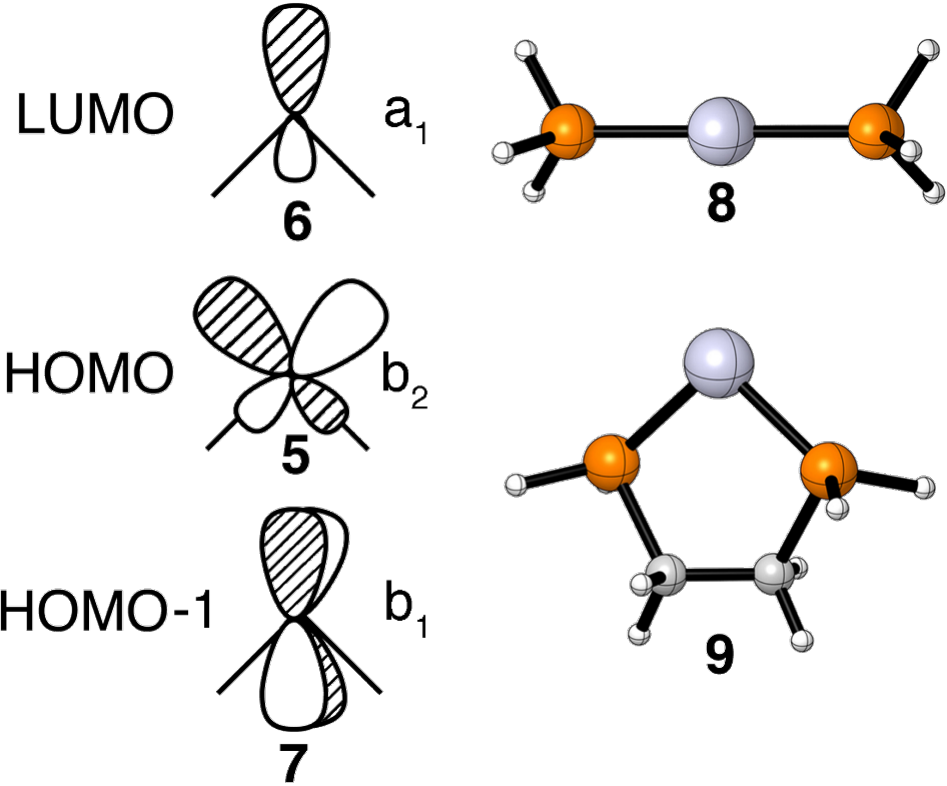
The important valence orbitals of a d^10^ ML_2_ group, **5**–**7**, along with the computed structures of Pt(PH_3_)_2_ and Pt(dpe).

A d^10^ ML_2_ fragment possesses a high-lying HOMO, shown by **5** in Figure 2, which has b_2_ symmetry and a low-lying LUMO, **6**, of a_1_ symmetry [22]. An energetically favorable reaction path will be one that maximizes the interactions of these orbitals with the orbitals of a coordinated polyene. The lowest occupied polyene π level is fully symmetric and, therefore, **6** can always interact with it. On the other hand, the LUMO in the π system may not always have the correct symmetry to interact with the b_2_ orbital on ML_2_ and it is the evolution of this overlap that has an important impact on the reaction path and activation energy. We will also have an occasion to consider a lower lying filled orbital of b_1_ symmetry, **7**.

Polyene–ML_2_ complexes are very fragile which in turn makes it somewhat difficult to compute the reaction path. The bond dissociation energy for ethylene–Pt(PH_3_)_2_ is only about 17 kcal/mol [23]. There are two ways in which the metal–polyene bond can be strengthened. The electron affinity for C_6_F_6_ is much larger than that for benzene [24]. Consequently interaction of the filled b_2_ fragment orbital with the LUMO of C_6_F_6_ is expected to be larger and the binding energy larger than that for benzene. The M and L that we shall use in this work is Pt and a phosphine. The second method employs the use of a bidentate phosphine. In this regard we have chosen diphosphinylethane (dpe). This idea here is that the P–Pt–P angle is around 100° in polyene–ML_2_ complexes. Upon dissociation the 14 electron PtL_2_ complex strongly prefers to be linear [22]. So the computed ground state for Pt(PH_3_)_2_, shown in **8**, is calculated to be 29 kcal/mol more stable than one where the P–Pt–P bond angle was constrained to be 99°. This of course is not the case for Pt(dpe), **9**. The P–Pt–P angle remains at 98°. Thus, the bond dissociation energy in polyene–Pt(dpe) complexes rises along with the attendant barriers for haptotropic rearrangements. This has been analyzed and quantified in detail by Massera and Frenking [23] for olefin–ML_2_ compounds.

## Computational Details

All geometries for the L = PH_3_ complexes were optimized without symmetry constraints within the DFT framework first using the B3LYP functional [25–27] in combination with the LANLDZ2 [28] basis sets. Single point calculations were carried out using the triple zeta d plus f polarization functions on Pt [29]. The geometry optimizations were then repeated using the M06 functional [30] along with the Def2-SV(P) basis set [31] for Pt, C, H and P except that the d functions on C were left off. Single point calculations used the Def2-TZVP basis [31] on Pt, P, C and H except for removing the f functions on C. F used a 6-31G basis [32] for the geometrical optimizations and 6-311G [33] in the single point calculations. Analytical frequencies were computed to determinate the nature of the stationary points. The Gaussian 09 software suite [34] was used in all of the calculations. The plots of the molecular structures utilized CYLview [35]. For brevity we will report the structures and Gibbs free energy differences in the standard state only for the polyene–Pt(dpe) complexes using frequencies from the Def2-SV(P) optimizations for the corrections to the Def2-TZVP energies. The geometries and total electronic energies are given as Supporting Information File 1.

## Results and Discussion

### A. Cyclic polyene–Pt(dpe) examples

**Figure 3:**
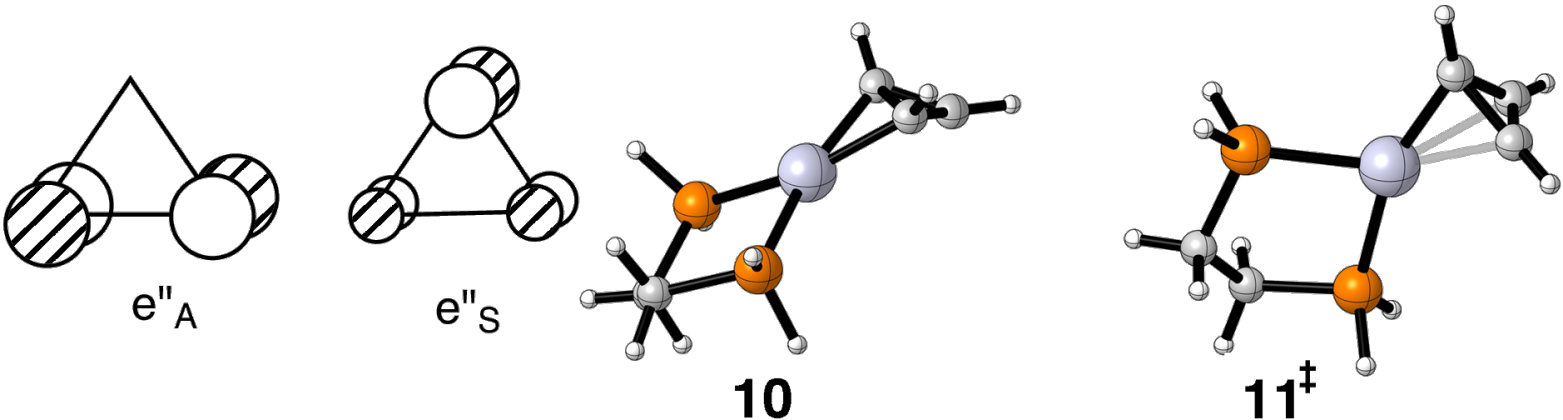
The empty degenerate set of π orbitals in the cyclopropenium cation is shown on the left side. On the right are the two optimized structures of C_3_H_3_–Pt(dpe)^+^.

The most simple of the cyclic polyenes is the cyclopropenium cation. Its LUMO is a degenerate par of π orbitals, labeled e”_A_ and e”_S_ in Figure 3. It is easy to see that e”_A_ interacts with the b_2_ orbital of ML_2_ at an η^2^ geometry. Indeed this is the computed group state for C_3_H_3_–Pt(dpe)^+^ as shown from a side view, **10**, in Figure 3. The transition state for shifting Pt(dpe) from one C–C bond to another passes through a geometry very close to η ^3^, as shown by **11**. Here b_2_ interacts with e”_S_ and along the reaction path a combination of the e” degenerate set. The essential features can be found elsewhere [21]. The Gibbs free energy difference between the two structures is small: 4.1 kcal/mol (2.4 kcal/mol for L = PH_3_). This is in accord with four structures of (Ph_3_C_3_)M(PPh_3_)_2_^+^ X^−^ where M = Ni, Pd, and Pt and X^−^ = ClO_4_ and PF_6_, which show a progressive movement of the ML_2_ unit over the face of the cyclopropenium ring [36]. These structures serve to chart this reaction path and this is consistent with a small reaction barrier with the resultant structure being determined by crystal packing effects. The details have been reported previously [21,36]. The optimizations reveal that the coordinated C–C bond is much longer, 1.62 Å, than the other two, 1.38 Å. This compares favorably to the M = Pt, X^−^ = PF_6_ structure [37] where the C–C distances are 1.58(2) and 1.39 Å, respectively.

**Figure 4:**
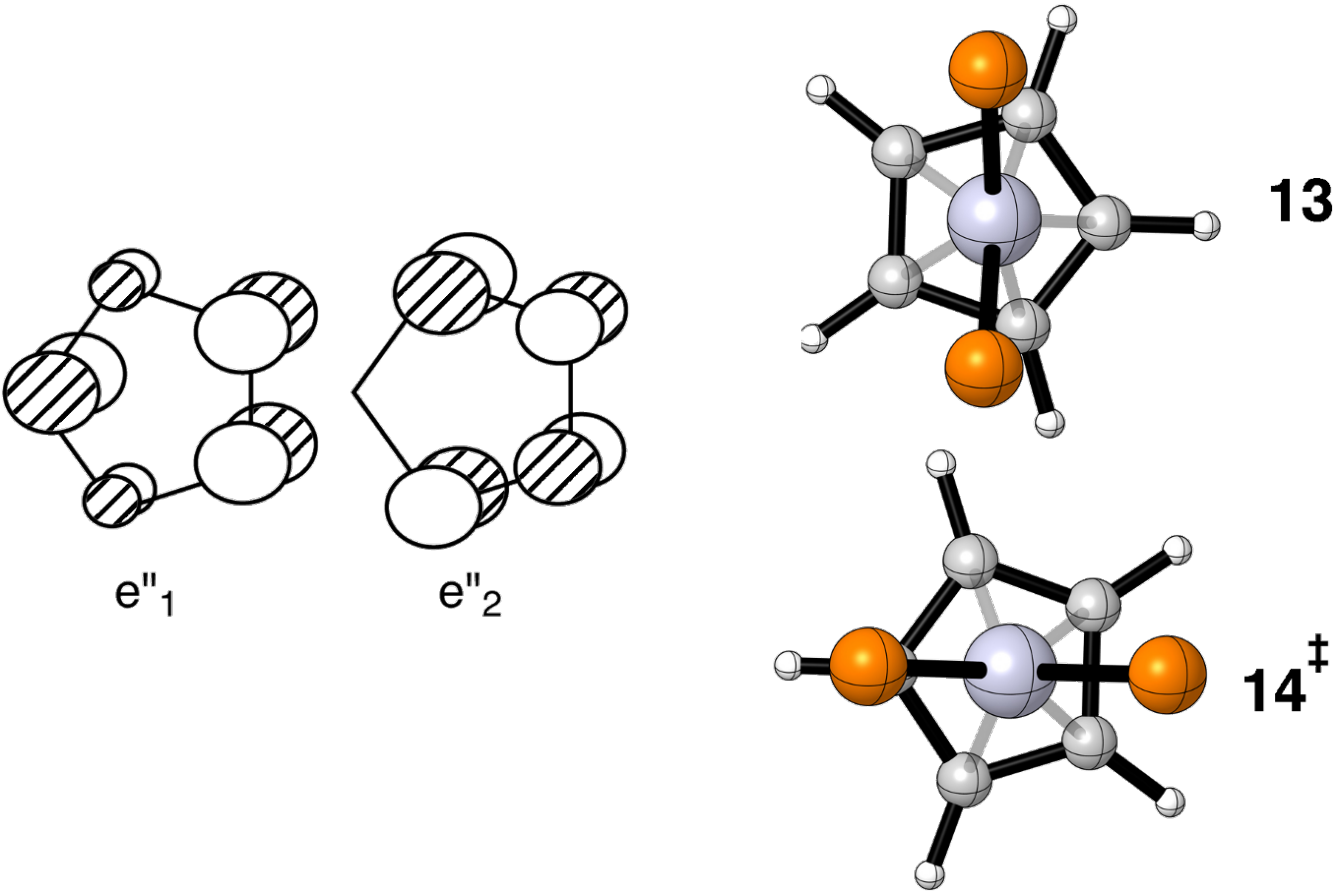
Two unoccupied MOs for Cp^+^ are shown on the left side. The two stationary points for Cp–Pt(dpe)^+^ are given by **13** and **14**. To conserve space the groups around the phosphorus atoms have been removed.

The situation for Cp–Pt(dpe)^+^ is very similar to the cyclopropenium case. Counting this as Cp^+^ means that there are two unoccupied orbitals that the b_2_ HOMO on ML_2_ can interact with. Each is one member of a degenerate set and they are shown on the left side of Figure 4. The two stationary points on the potential energy surface are displayed from a top view on the right side of Figure 4. The e”_2_ fragment orbital can interact with b_2_ to form an η^3^ complex as shown in **13**. An η^5^ geometry, **14**, will be favored using the empty e”_1_ orbital. The computed Gibbs free energy difference between the two is very small, namely 1.5 kcal/mol favoring η^3^. A recent search of the Cambridge crystallographic database [38] reveals 29 structures of the Cp- and indenyl-M(PR_3_)_2_^+^ type where M = Ni, Pd, Pt. For the more general CpML_2_ case where M = Fe through Pt there are 1074 hits. The majority of these structures are close to the η^5^ type although most have a significant range of M–C bond distances. For example, in cyclopentadienyl-platinum-bis(diphenylphosphinobiphenyl) [39] there are two Pt–C distances at 2.26(1) Å and one at 2.33(1) Å. The conformation of the PtL_2_ unit with respect to the Cp ring is approximately that given by **13**. Accordingly, the remaining two Pt–C distances are 2.37(1) Å. For optimized **13** the corresponding set of distances is 2.29, 2.34 and 2.45 Å, respectively. The indenyl-M(PR_3_)_2_^+^ examples are decidedly η^3^ as a consequence of the perturbation generated by the benzo substituent. Normally one would do the electron counting in these molecules as Cp^−^ and d^8^ ML_2_ yielding an 18-electron complex. The b_2_ fragment orbital is now formally empty and the e”_1_ set is filled. A full discussion of the bonding in these compounds may be found elsewhere [22].

**Figure 5:**
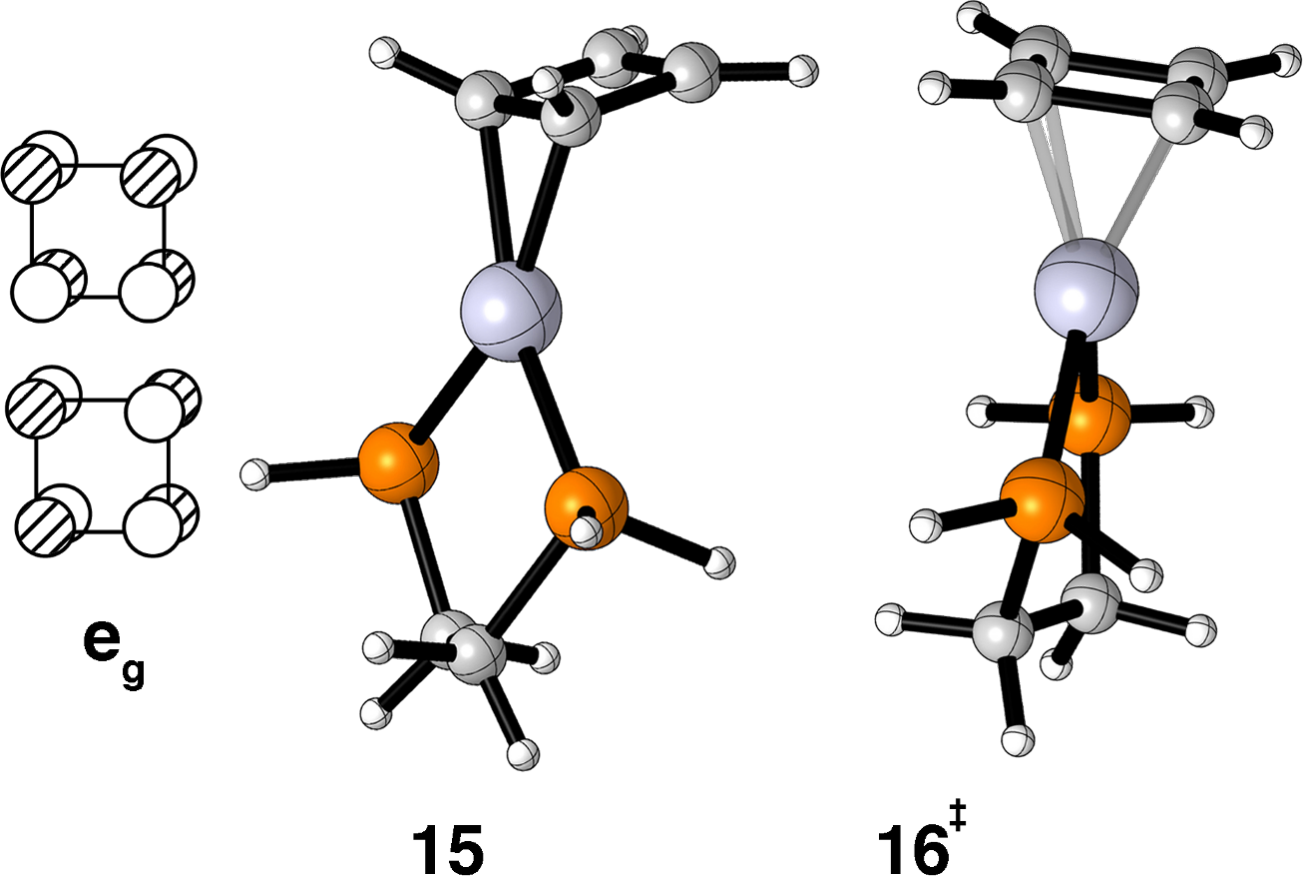
The half-filled degenerate π orbitals in cyclobutadiene. The computed ground state (**15**) and transition state (**16**) for cyclobutadiene–Pt(dpe) on the right.

Another polyene with two coordination geometries is cyclobutadiene. The e_g_ set shown on the left side of Figure 5 is half-filled. It is easy to see that one member has the correct symmetry to interact with b_2_ ML_2_ at both the η^2^ and η^4^ geometries. We found for cyclobutadiene–Pt(dpe) that the η^2^ geometry, **15**, is 6.5 kcal/mol more stable than the η^4^ geometry, **16**. For L = PH_3_ the energy difference is even larger, 10.5 kcal/mol. These results are a little surprising in that the energy difference is larger than what we expected. We are aware of only one structure at this electron count, Ph_4_C_4_–Ni(PEt_3_)_2_ [40], and it is clearly η^4^. As we shall see later, the difference between Ni and Pt can be significant but for the time being, experiment and theory are not in agreement with each other.

**Figure 6:**
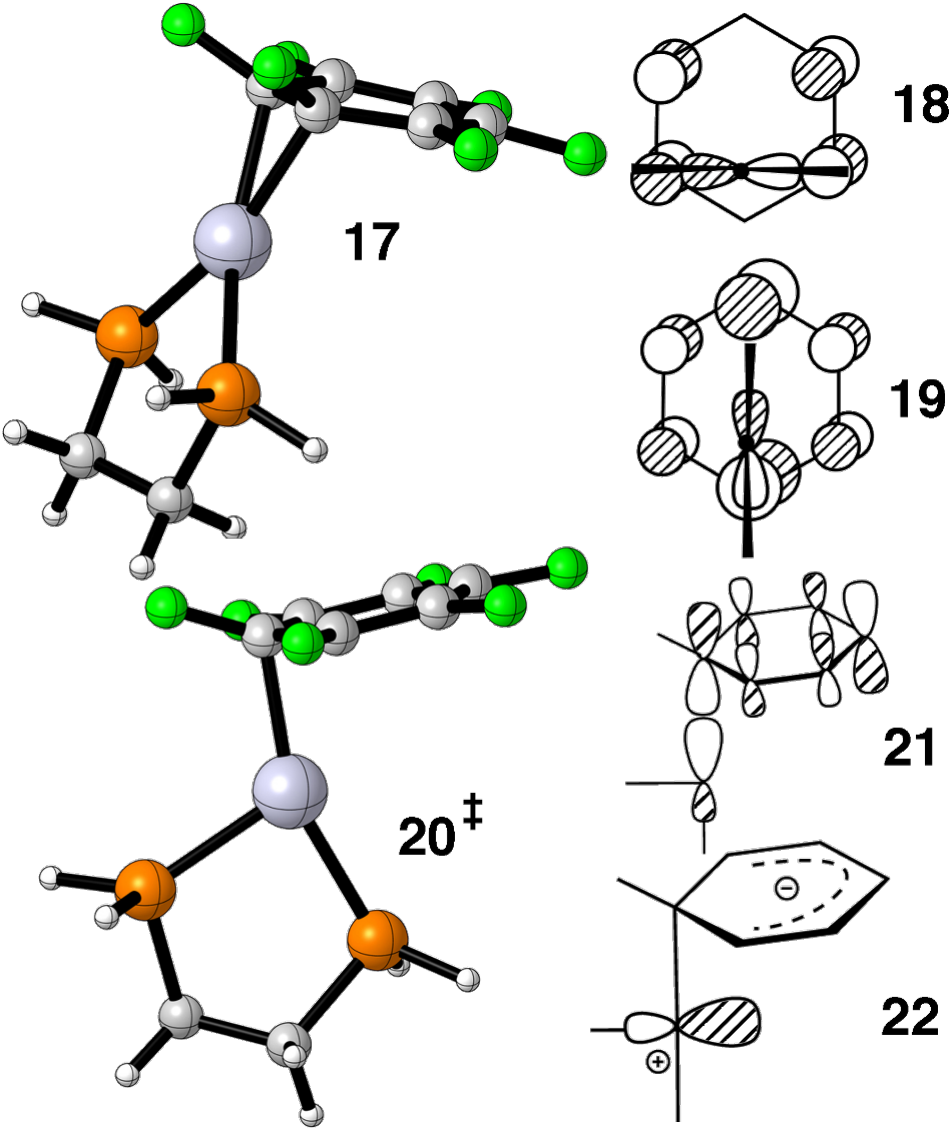
The ground and transition state for ring whizzing in F_6_C_6_–Pt(dpe), **17** and **20**, respectively. The dominant bonding interaction for two possible transition states, **18** and **19** along with the HOMO, **21** and LUMO, **22**, in the η^1^ transition state.

Benzene–Ni(PR_3_)_2_ compounds have been known for some time [41]. An η^2^ geometry has been observed to be the precursor to C–F bond insertion for F_6_C_6_ complexes [42] and a number of theoretical studies have been carried out [43–46] which address this reaction. There are two arene–Pt(PR_3_)_2_ structures in the literature [47–48] and both have η^2^ geometries. The barrier for ring whizzing in (CF_3_)_6_C_6_–Pt(PEt_3_)_2_ has been measured to be ≈11 kcal/mol [41]. One member of the LUMO e_1g_ set in benzene has a large overlap with the b_2_ ML_2_ MO. The computed ground state structure for η^2^ F_6_C_6_–Pt(dpe), **17** in Figure 6 agrees well with the experiment. The issue is whether the transition state for ring whizzing favors the interaction between e_1g_ and b_2_ shown from a top view in **18** or **19**. Extended Hückel calculations favored the former [20–21]. Our present day calculations, however, favor **19**. The structure is shown in **20**. Special care was taken to search for a transition state where the Pt(dpe) group was rotated by 90° but none was found. The activation barrier was computed to be 7.4 kcal/mol. Reinhold, McGrady and Perutz [46] obtained a barrier of 6.4 kcal/mol for the same molecule using the B3LYP hybrid functional and a different basis set. The computed geometric parameters for the molecules are very close to each other. One Pt–C bond is short (2.10 Å) while the other two flanking bonds are 2.52 Å. Thus, **20** strongly resembles an η^1^ 14 electron complex with a “T” shaped geometry. An easy way to view these results is to take a linear combination of b_2_ (**5**) and a_1_ (**6**). This will generate two equivalent dsp hybrids. One will be filled and can interact with one component of the e_1g_ LUMO, **21**, in Figure 6 and the other will remain empty, **22**.

Another highly fluxional molecule is cycloheptatrienyl–Pt(dpe)^+^ which exhibits a situation similar to that described for Cp–Pt(dpe)^+^. The ground state is again an η^3^ structure. This is in agreement with several substituted cyclohepatrieneyl–PdL_2_ complexes [49]. We looked hard for an η^5^ species but instead found an η^2^ structure which serves as a transition state for ring whizzing. The activation barrier was computed to be 3.2 kcal/mol. Barriers from 10.5 to 7.6 kcal/mol were found for the Pd complexes [49]. Interestingly an η^1^ transition structure with one imaginary frequency was also discovered. It was found to be 7.2 kcal/mol above the ground state.

**Figure 7:**
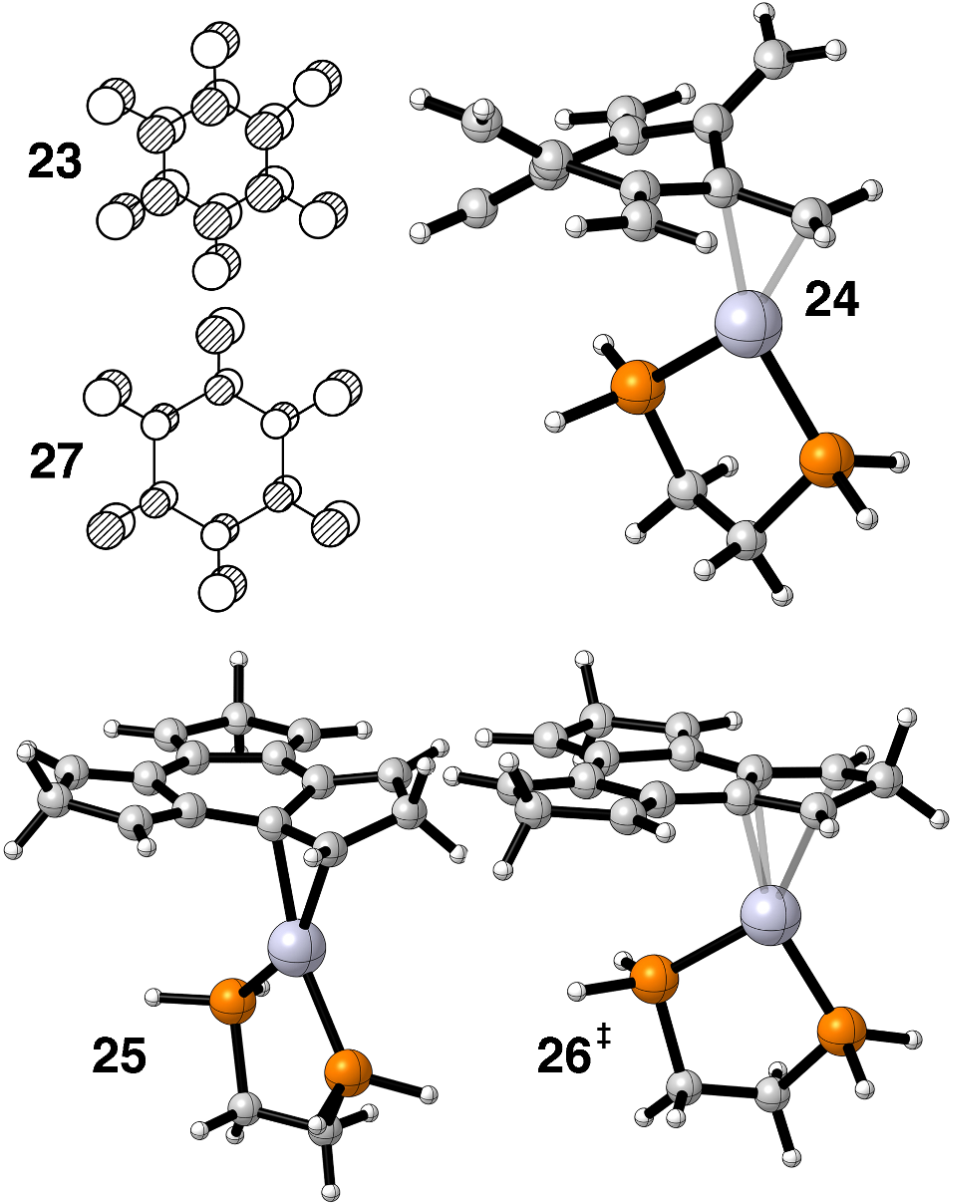
The LUMO, **23**, and HOMO, **27**, in 6-radialene. The optimized η^2^ ground states are shown in **24** and **25** while **26** shows the geometry for one η^4^ transition state.

We thought that radialenes would be an attractive candidate as a ligand and would exhibit a facile haptotropic rearrangement when coordinated to Pt(dpe). The LUMO is all-in phase combination of olefinic π* as shown for 6-radialene by **23** in Figure 7. Therefore, the ML_2_ b_2_ fragment would retain a sizable portion of its overlap on going from an η^2^ to η^4^ geometry. For some time 6-radialene and many alkyl derivatives have been known [50]. It is extraordinarily reactive and a bis-Fe(CO)_3_ derivative of 5-radialene has recently been prepared [51]. The structure of 6-radialene is strongly distorted into a chair form with a boat conformation slightly higher in energy [51]. The *D*_6_*_h_* structure lies higher in energy by 17.1 kcal/mol [51]. Our optimization of the η^2^ ground state shows a twisted boat conformation to be the most stable, **24**, in Figure 7. The activation barrier was found to be 13.7 kcal/mol. We thought that by tying the ends of the olefins together via a CH_2_ group would force the ligand to be flat. In fact there are compounds analogous to this having O, S and Se as the linker that are in fact flat [52]. Our calculations reveal that the η^2^ ground state, **25**, and the η^4^ transition state, **26**, are essentially flat, but the energy difference is only lowered to 13.0 kcal/mol. In **25** the two Pt–C bond distances are 2.17 Å, however, in **26** they are considerably lengthened. The inner Pt–C distances are 2.36 Å and the ones adjacent to the CH_2_ group are 2.61 Å! The principal destabilization in **26** is due to the interaction between b_1_ (**7**) and the HOMO on 6-radialene, which is the totally antibonding combination of π orbitals, **27**.

### B. The strange case of cyclooctatetraene

**Figure 8:**
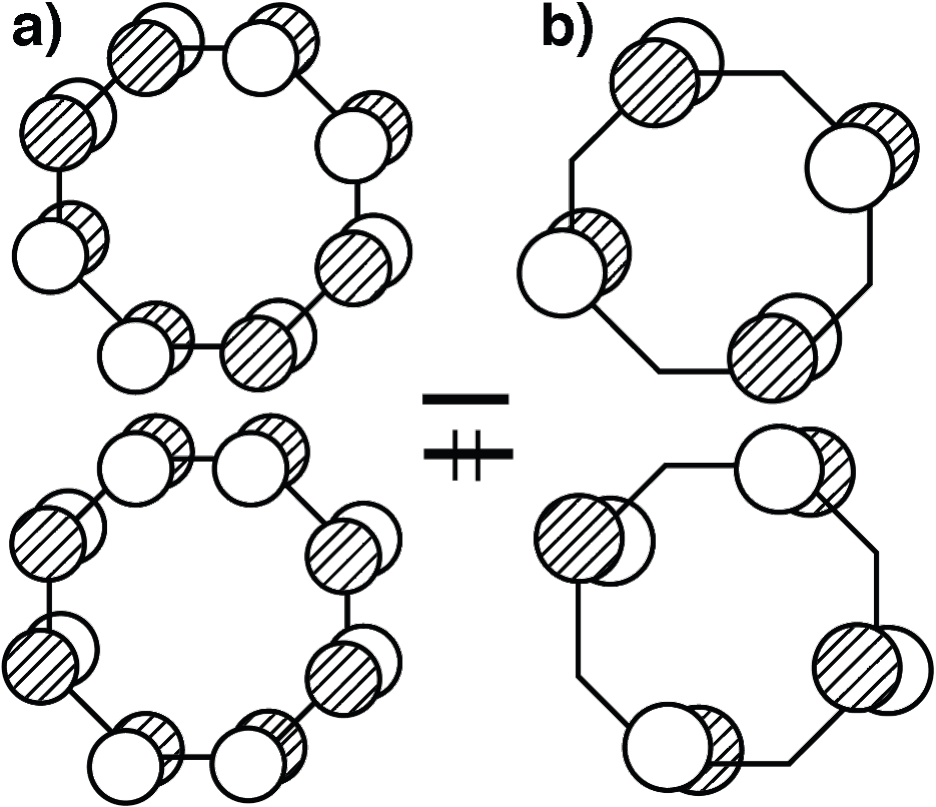
Two representations for the half-filled e_2u_ set of π orbitals in cyclooctatetraene.

**Figure 9:**
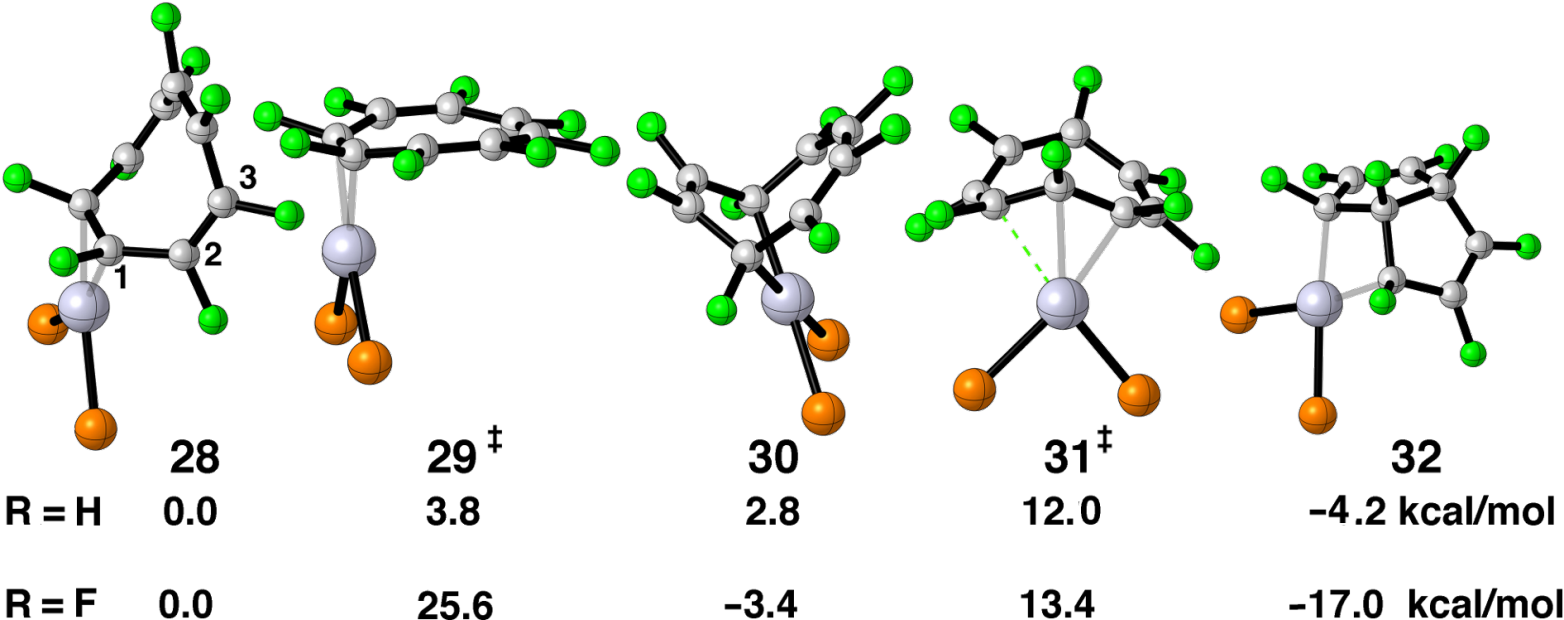
The stationary points found on the potential energy surface of C_8_F_8_–Pt(dpe). For clarity the groups around the phosphines have been removed. The relative energies for this compound, as well as COT–Pt(dpe) are given below each structure.

**Figure 10:**
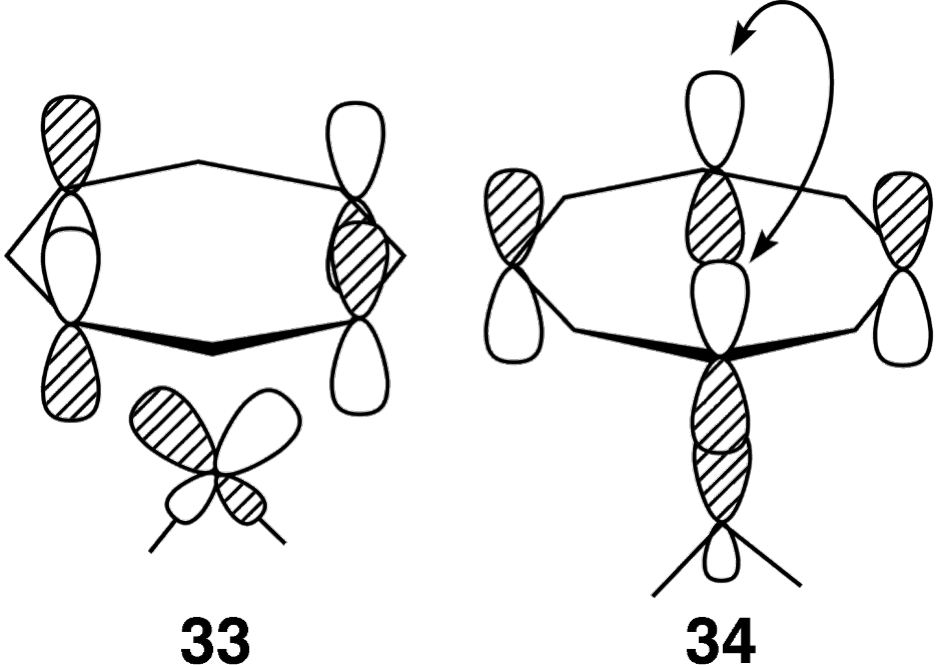
The two important bonding interactions for transition state **31** are drawn in **33** and **34**.

Cyclooctatetraene (COT) has been a favorite ligand since the dawn of organometallic chemistry [2]. Figure 8 shows two representations for the half-filled e_2u_ set of π orbitals in the flat *D*_8_*_h_* geometry. One can see from the representation in a) that an η^2^ or η^4^ conformation are possibilities. In b) one can envision η^1^ or η^3^ as potential structures. The optimized structures for C_8_F_8_–Pt(dpe) are illustrated in Figure 9. To conserve space the groups around the phosphorus atoms have been removed. COT and C_8_F_8_ have a tub shaped structure with *D*_2_*_d_* symmetry [53–54]. As expected an η^2^ structure, **28**, was found to be a minimum. A 1,4-diyl minimum was also found where there are two Pt–C σ bonds, **30**. This structure has also been suggested by means of the low temperature ^31^P and ^13^C NMR of COT-Pt(R_2_PCH_2_CH_2_PR_2_), R = iPr [55]. The transition state that interconnects **28** to **30** is shown in **29**. The coordination geometry around Pt is typical of that in η^2^ olefin complexes. What is novel is that the COT (and C_8_F_8_) ring is essentially flat with the uncoordinated portion of the polyene having alternating C–C bond lengths of ≈1.45 and 1.35 Å. This is in fact the structure of an analogous Ni complex as determined by X-ray crystallography [56]. The haptotropic rearrangement of **28** to **30** does not permute all of the carbon atoms in the COT ring. There is a mirror plane in the plane of the paper for all of the structures in Figure 9. This equivalences the carbons on the front side of the paper with those on the back side. Compounds **28**–**30** do not have a mirror plane perpendicular to this and, therefore, C2 (see **28**) does not become equivalent to C3, etc. As we shall see, a structure akin to **35** would accomplish this. In searching for another structure that accomplishes this we discovered tricyclic **32**. The transition state that converts **28** into **32** is **31**. For the C_8_F_8_ complex, **28**, the Pt–C distances are 2.08 Å. In **31** the corresponding distances are 2.11 and 2.26 Å with the dashed green bond being formed measuring at 2.32 Å. In COT–Pt(dpe) the transition state **31** is akin to an η^3^ complex with the three Pt–C bond lengths calculated to be 2.22–2.26 Å. Since **32** has *C*_s_ symmetry (discounting the dpe ligand), it serves as a way-point for ring-whizzing. It is easy to see the electronic basis for ring folding and construction of the tricyclic molecule. Consider that in **28** the filled ML_2_ b_2_ orbital coordinates to the two lower p AOs in the upper component of e_2u_ in Figure 8a. Then empty a_1_ interacts with the lower component in Figure 8a. As ML_2_ slips over the polyene in a clockwise motion the appropriate e_2u_ representations become those in Figure 8b. The empty orbital at the top right in Figure 8 interacts with the filled b_2_ ML_2_ orbital and a_1_ interacts with the filled e_2u_. This is explicitly drawn in **33** and **34**, respectively, of Figure 10. The important consequence of this motion is that the p AO on the opposite side of the ring in **34** has the correct phase to generate a C–C σ bond and this collapses to bicyclic **32**.

Our calculations find that C_8_F_8_–Pt(dpe) will be caught in the deep potential energy well of the tricyclic isomer, **32**. Hughes and co-workers have shown that experimentally this is indeed the case [57–58]. With PPh_3_ and AsPh_3_ ligands compounds analogous to **30** are initially formed from the reaction of C_8_F_8_ and a Pt(0) precursor. **30** then irreversibly rearranges in solution to **32** overnight at room temperature. This is also in accord with our calculations. Notice that going from **30** to **32** requires the passage through transition state **29**, which requires 29 kcal/mol. We think that the reason why **29** lies much higher in energy than the COT analog is due to the energy cost associated with flattening the ligand to a *D*_4_*_h_* type of geometry. For COT itself this entails an energy cost of 10–13 kcal/mol [59]. We find that the conversion for C_8_F_8_ is nearly triple this amount, namely 29.9 kcal/mol [60].

**Figure 11:**
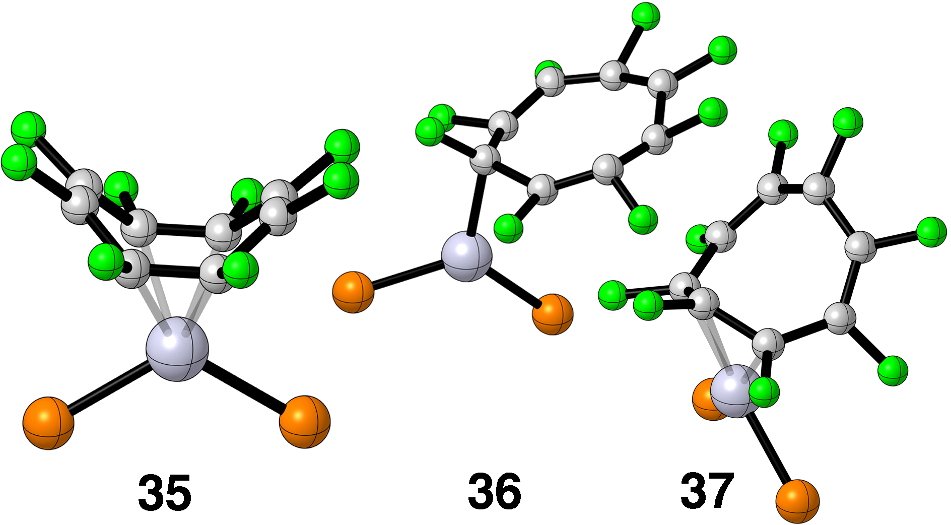
Three other coordination geometries that did not lead to new stationary points are shown in **35**–**37**.

The picture for COT–Pt(dpe) is not so clear. Our calculations would have **28**, **30** and **32** in rapid equilibrium with the overwhelming majority of the equilibrium shifted to the tricyclic compound. The low temperature ^31^P and ^13^C NMR of COT–Pt(R_2_PCH_2_CH_2_PR_2_), R = iPr [55], clearly shows that either **28** or **31** (the authors prefer **31**) is in rapid equilibrium with **30**. There is no spectroscopic evidence consistent with the existence of **32**. It may well be the case that bulky iPr groups in place of hydrogens alter the relative energetics. Perhaps computations with a different functional and/or a larger basis set might bring theory and experiment into agreement. Furthermore, moving from Pt to the isoelectronic Ni also can have a significant impact. An X-ray of the COT–Ni complex [56] reveals the structure is analogous to that for **29**. An X-ray of another Ni complex [60] produces a bis-η^2^ isomer, **35**. This is also true for C_8_F_8_–Ni complexes with certain ligand sets [57–58]. We carried out a number of potential energy minimizations as shown in Figure 11 starting from **35**, as well as, η^1^, **36**, and η^3^, **37**. Unfortunately none of these produced new stationary points. We will return to this Ni versus Pt issue later.

### C. Polycyclic examples

**Figure 12:**
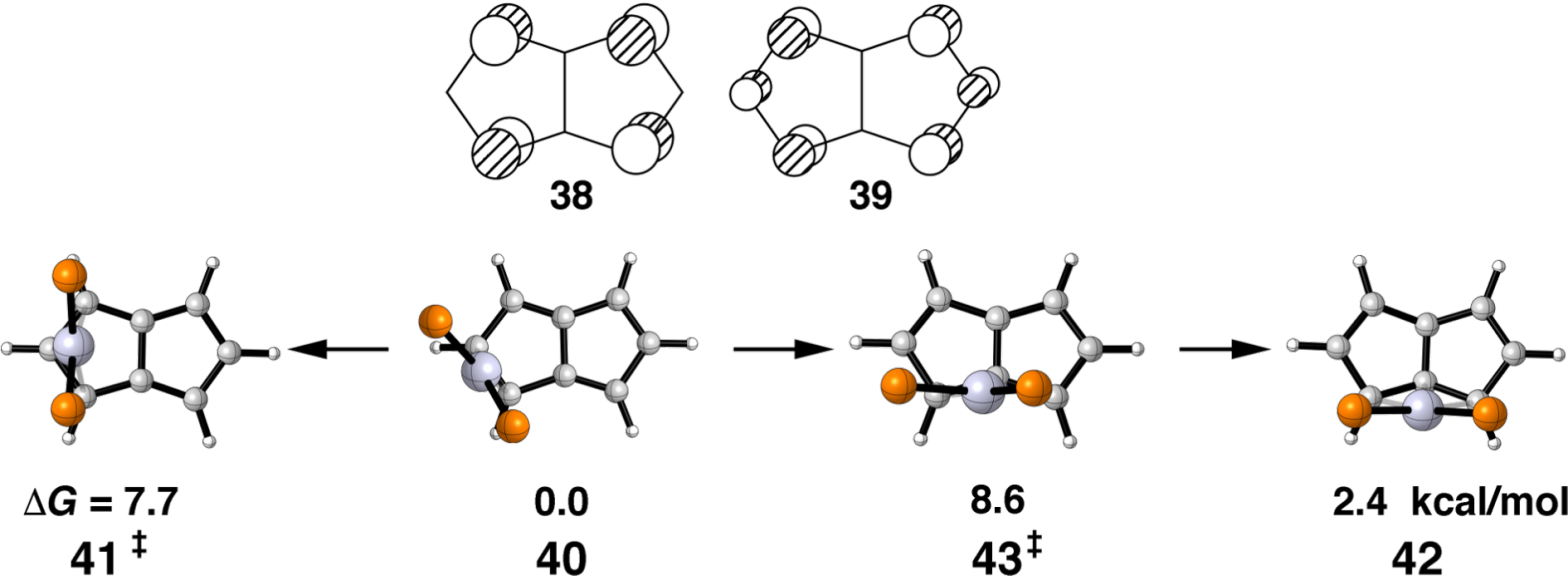
The LUMO and LUMO+1 shown in **38** and **39**, respectively. The four stationary points found for pentalene–Pt(dpe) are displayed in **40**–**43** along with their relative energies. The groups connected to the phosphorus atoms are not shown.

Pentalene metal complexes have been the subject of a number of investigations [61], as well as, theoretical explorations of haptotropic rearrangements with ML_3_ and MCp [62–63]. However, we are not aware of any complexes with a d^10^ ML_2_ group. Pentalene has an energetically low-lying LUMO and close to it another empty orbital. These are shown in **38** and **39**, respectively, in Figure 12. It is easy to see that in **38** the b_2_ ML_2_ fragment orbital can interact in an η^3^ mode both within the five-membered ring, as well as, between the two. **39** has the correct topology to interact with b_2_ in η^2^ and η^3^ modes. We were able to locate four stationary points on the potential energy surface of pentalene–Pt(dpe). These are shown from a top view along with their relative energies in Figure 12. Here again the hydrogens and ethano-bridge connected to the phosphorus atoms has been removed for clarity. We find that the η^2^ structure, **40**, to be the ground state. A low energy η^3^ transition state, **41**, at 7.7 kcal/mol serves to equivalence the top and bottom halves of the pentalene ligand. The Pt(dpe) group can migrate from one ring to the other via the η^3^ structure, **42**. Again the activation energy associated with the transition state **43** is predicted to be small at 8.6 kcal/mol. We anticipate that pentalene–Pt(PR_3_)_2_ will be a highly fluxional molecule.

**Figure 13:**
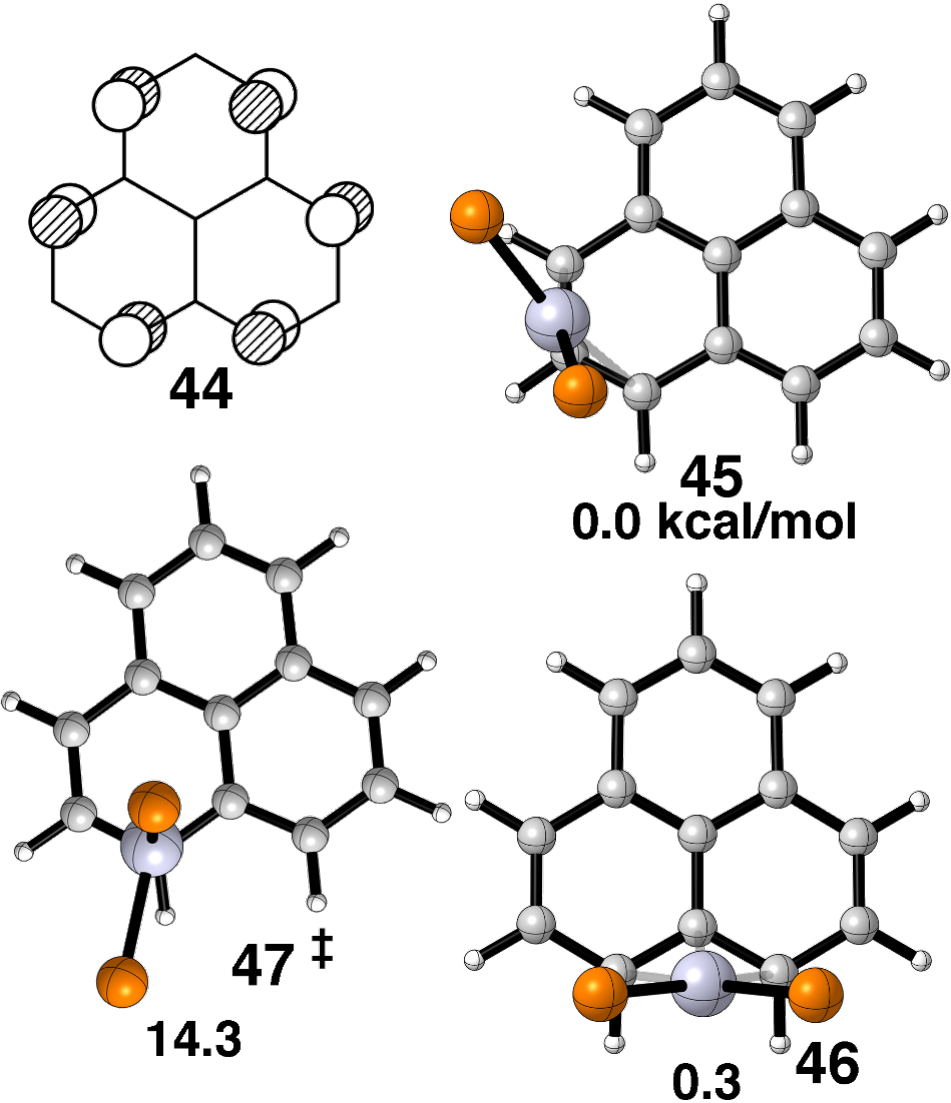
The LUMO of the phenalenium cation is given in **44**. The structures of the three stationary points found for phenalenium–Pt(dpe)^+^ along with their relative energies are shown from a top view in **45**–**47**. Again the groups connected to the phosphorus atoms are not shown.

The situation for phenalenium–Pt(dpe)^+^ is very similar. The LUMO for phenalenium^+^ is a rigorously non-bonding MO, **44** in Figure 13. One expects and finds η^3^ structures both within and between rings as given by **45** and **46**, respectively, with essentially identical relative energies. Experimentally, all known complexes [64–67] are akin to **45**. Our calculated barrier of 14.7 kcal/mol via **47** seems a bit too low. The measured barrier in two Pd(tmeda) complexes was 21.4 and 21.6 kcal/mol [66]. No signs of fluxionality was found in a substituted phenalenium–Pt(PPh_3_)_2_^+^ complex [67].

**Figure 14:**
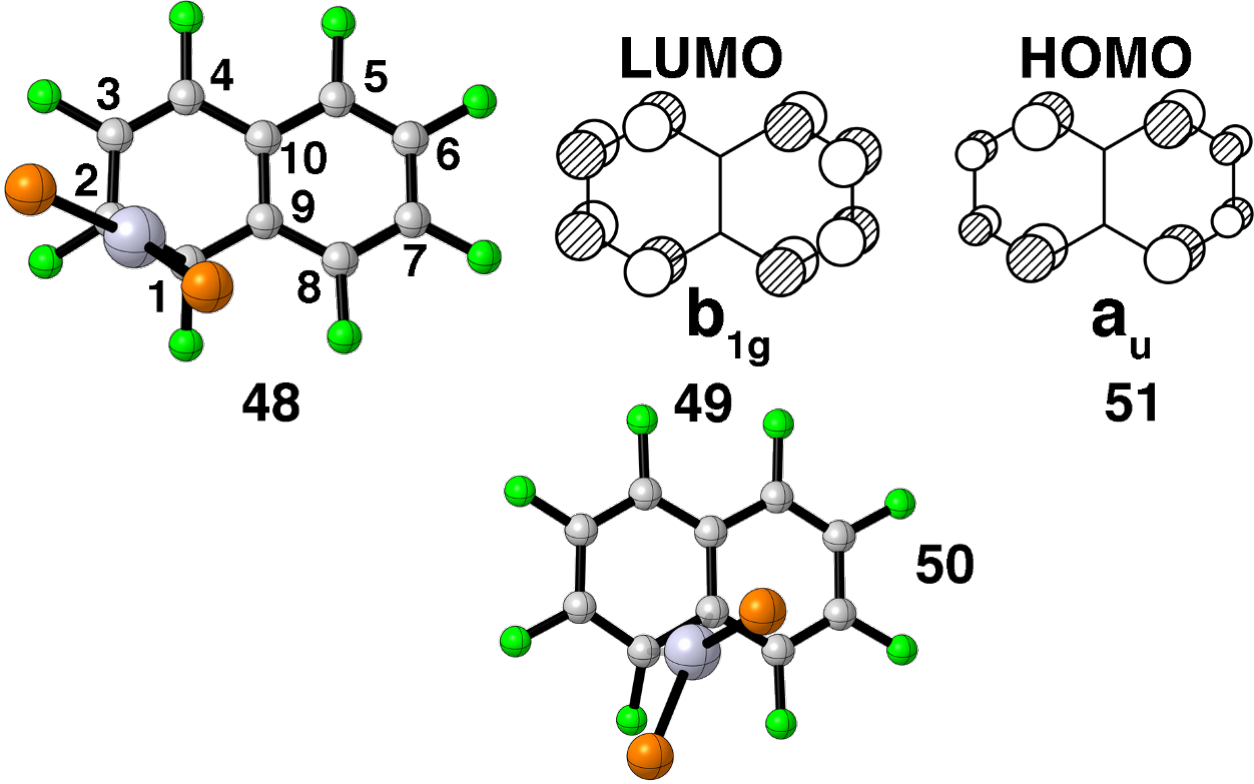
A top view of two stationary points found for F_8_C_10_–Pt(dpe); **48** is the ground state and **50**, represents one point on the plateau. The LUMO and HOMO in naphthalene are drawn in **49** and **51**, respectively.

Naphthalene and anthracene–Ni(PR_3_)_2_ compounds have been known and studied for some time [45–46,68–75]. We are, however, unaware of any Pt(PR_3_)_2_ examples. The ground state structures of the Ni compounds possess an η^2^ geometry where the Ni is coordinated to a carbon–carbon bond adjacent to the ring fusion. Our calculations on octafluoronaphthalene–Ni(dpe) and –Pt(dpe) (as well as naphthalene–Pt(dpe) itself) are in good agreement with experiment. A top view of the structure is shown by **48** in Figure 14. This offers a good overlap between the LUMO in C_10_F_8_, **49**, and the b_2_ HOMO, **5**, in Pt(dpe). It was thought [20] that migration of an ML_2_ unit from one ring to another would involve an η^3^ structure where Pt would bond to C(1), C(9) and C(8). For the carbon numbering system please see **48**. Bonding between b_2_ ML_2_ and the b_1g_ MO would be retained. Unfortunately this is not quite the entire story. One of the stationary points is shown by **50**. The a_u_ HOMO, **51**, in C_10_F_8_ also has a significant overlap with b_2_ at this geometry. Since these two fragment orbitals are both filled, there is also considerable destabilization. What we find is that this expanse of the potential energy surface is a twixtyl intermediate [76]. At the stationary point given by **50** there is one imaginary frequency of 17i cm^−1^; at another closer to η^3^ the computed frequencies are all positive but one is tiny, 15 cm^−1^. So this region of the coordinate space is analogous to a plateau; the potential energy is essentially flat. The activation energy to attain **50** in C_10_F_8_–Pt(dpe) was computed to be 13.7 kcal/mol; in C_10_H_8_–Pt(dpe) the barrier was 14.8 kcal/mol. This is in line with an NMR derived barrier of about 15 kcal/mol for C_10_H_8_NiL_2_ [74] and 15–20 kcal/mol for anthracene–Ni(PR_3_)_2_ [69–70]. Oprunenko and Gloriozov [75] have calculated the η^3^ tranisition state to lie at a relative energy of 12.2 kcal/mol for naphthalene–Ni(PEt_3_)_2_ using the PBE functional and a different basis set than that employed here. Jones and co-workers [45] have undertaken an exhaustive study of ring whizzing and oxidative addition in a series of cyano and methyl substituted naphthalene–Ni(dmpe) complexes at the B3LYP level. Structures analogous to **50** were reported at relative energies of 12–17.5 kcal/mol. We do find in C_10_F_8_–Pt(dpe) that there is a second path for the haptotropic rearrangement from one ring to the other. Here the Pt(dpe) group migrates further in towards the ring junction with a weakly bound transition state of 21.9 kcal/mol and ending at an η^2^ minimium where the C(9) and C(10) atoms are coordinated to Pt at a relative energy of 17.9 kcal/mol. The latter structures were also computed to lie at high energies by Jones and co-workers [45]. So, at this point theory and experiment appear to be in agreement for the NiL_2_ and PtL_2_ cases.

**Figure 15:**
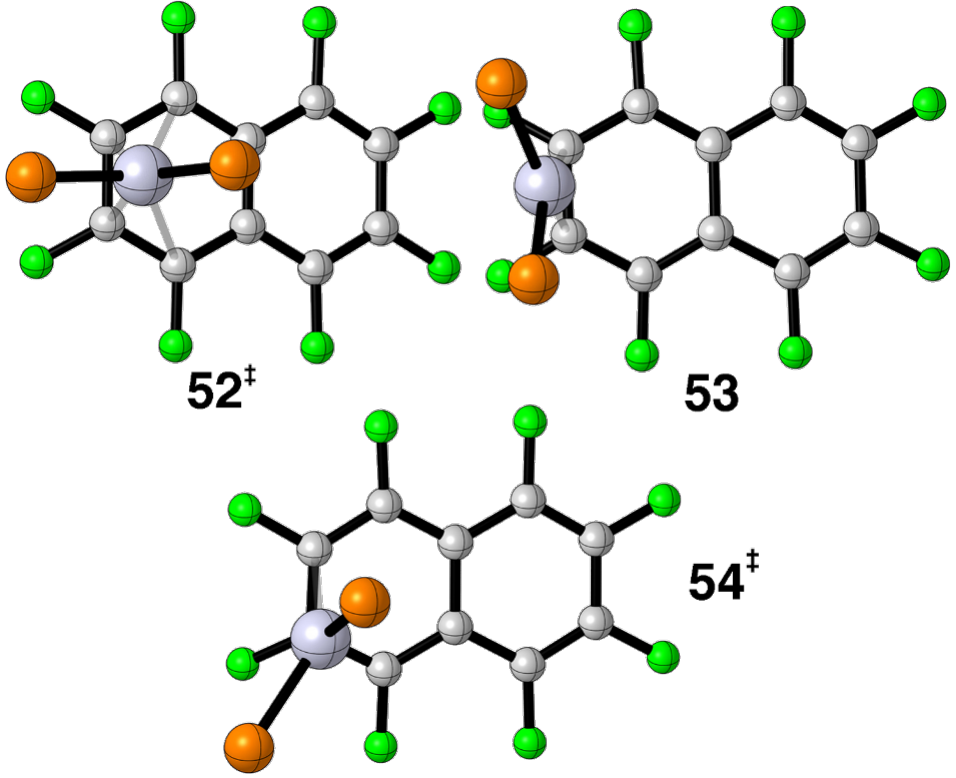
At top view of the η^4^, **52**, and η^4^, **54**, transition states along with the η^2^, **53**, intermediate.

But the story does not end here. Experimentally there is a low energy process that converts, **48**, to the equivalent η^2^ complex where the ML_2_ group is coordinated to C(3) and C(4). This is also the case for anthracene–NiL_2_. The experimental barriers range ≈5–6 kcal/mol [69–70,74]. The aforementioned calculations [45,75] yield barriers of 4.2–9.5 kcal/mol in reasonable agreement with the experiment. The structures of these transition states resemble η^4^ species with the geometry akin to **52** in Figure 15. This is not the case for C_10_F_8_–Pt(dpe) or C_10_H_8_–Pt(dpe). The barriers are calculated to be 17.1 and 17.4 kcal/mol, respectively. Furthermore, the barrier for C_10_H_8_–Pt(dpe) using the B3LYP function generates a barrier of 17.8 kcal/mol. Using the M06 functional for C_10_F_8_–Ni(dpe) yields a barrier of 6.1 kcal/mol which is in line with the calculations by others. Therefore, the discrepancy must lie in the difference between Pt and Ni. There is also a difference in the metrical details of these transition states. For the Ni examples the Ni–C(1) and Ni–C(4) distances are ≈0.3 Å longer than the Ni–C(2) and Ni–(3) ones (see **48** for the numbering scheme). For the Pt complexes we find this difference to be about twice as large. In other words, the Pt cases are closer to η^2^ complexes where the olefinic portion of the ligand is rotated by 90° from the minimum energy conformation given in **1**. We will return to this point shortly. One might think that the overlap between b_2_ ML_2_ and the LUMO, **49**, from the η^2^ ground state to η^4^ will be retained and, thus, the activation energy will be small. However, note that at η^4^ the overlap between the filled b_1_ fragment orbital, **7**, and the a_u_ HOMO on C_10_F_8_ is turned on and this is repulsive. With this in mind it is tempting to put forward the hypothesis that the 3d AOs in Ni are very contracted and their overlap at **52** is not so large. Hence the a_u_ – b_1_ repulsion is not so large and it is the mixing of 4p character in the Ni b_2_ orbital that retains reasonable overlap with b_1g_. On the other hand, the Pt 5d AO is more diffuse and consequently more bonding is lost at η^4^ than its Ni congener. But this cannot be the whole story. Massera and Frenking [23] have shown that there is essentially no energy difference between the bond dissociation energy (BDE) in ethylene–Ni(dpe) and the Pt analog. Furthermore, their calculated BDE for ethylene–Pt(PH_3_)_2_ is in very good agreement with that found [23] at the CCSD(T) level with a large basis set. On the other hand, Reinhold, McGrady and Perutz have reported [46] that C_6_H_6_ and C_6_F_6_–Pt(dpe) BDEs are about 8 kcal/mol less than that for the Ni(dpe) analogs.

A close examination of the potential energy surface in C_10_F_8_–Pt(dpe) revealed the existence of another η^2^ minimum, **53**, in Figure 15. It lies 13.7 kcal/mol above the ground state. This is in line with the corresponding minima found by Jones and co-workers [45] in the substituted naphthalene–Ni(dmpe) compounds (≈13 kcal/mol). So our calculations put the η^2^ minimum, **53**, to be 3.4 kcal/mol more stable than the η^4^ transition state, **52**. However, the latter does not serve as the waypoint for the former. An η^1^ structure, **54**, was found to be the transition state for the haptotropic rearrangement of **48** to **53**. Notice that passage through the η^2^ intermediate causes the phosphines to become equivalent. Benn and co-workers [74] in fact observe phosphine equivalence with a barrier of approximately 13 kcal/mol for the naphthalene–Ni(PR_3_)_2_ compounds. Our calculations put **54** to be 14.9 kcal/mol above the ground state, **48**. This is in reasonable agreement with the NMR results [74]. The reaction path and associated electronic details for the **48** to **54** to **53** haptotropic shift is precisely analogous to ring-whizzing in C_6_F_6_–Pt(dpe) that was covered previously. In summary we find the potential energy surface for naphthalene–Pt and –Ni complexes to be quite different. In C_10_F_8_–Pt(dpe), haptotropic rearrangement from one ring to another is energetically similar to that within one ring, whereas, in the Ni analog the former is much slower than the latter.

## Conclusion

Our original thesis that the ML_2_ b_2_ interaction with the LUMO of the polyene dictated the reaction path was largely fulfilled. Often this guided our exploration of the potential energy surfaces. But molecules, like life, sometimes yield unexpected conclusions. We miss you, Peter Hofmann.

## Supporting Information

File 1 The molecular geometry and total electronic energy for the molecules in this work are given in .xyz format. The file may be opened as a text file to read the coordinates, or opened directly by a molecular modeling program such as Mercury (http://www.ccdc.cam.ac.uk/pages/Home.aspx).
Molecular geometry and total electronic energy data.
